# The Association of Planetary Health Diet with the Risk of Type 2 Diabetes and Related Complications: A Systematic Review

**DOI:** 10.3390/healthcare11081120

**Published:** 2023-04-13

**Authors:** Omorogieva Ojo, Yiqing Jiang, Osarhumwese Osaretin Ojo, Xiaohua Wang

**Affiliations:** 1 School of Health Sciences, Faculty of Education, Health and Human Sciences, University of Greenwich, London SE9 2UG, UK; 2 The School of Nursing, Soochow University, Suzhou 215006, China; 3 Smoking Cessation Department, University Hospital, South London and Maudsley NHS Foundation Trust, Lewisham High Street, London SE13 6LH, UK

**Keywords:** planetary health diet, EAT-Lancet diet, cardiovascular risk, risk of type 2 diabetes, obesity indicators, indicators of environmental sustainability

## Abstract

Background: Nutritional interventions such as the planetary health diet, which the EAT-Lancet commission proposed, may be an effective strategy for reducing type 2 diabetes risks and its associated complications. The planetary health diet demonstrates the significant role of diet in associating human health with environmental sustainability and the significance of transforming food systems in order to ensure that the UN’s Sustainable Development Goals and the Paris Agreement are achieved. Therefore, the aim of this review is to examine the association of the planetary health diet (PHD) with the risk of type 2 diabetes and its related complications. Method: The systematic review was conducted in line with established guidelines. The searches were carried out in health sciences research databases through EBSCOHost. The population, intervention, comparator and outcomes framework was used in order to define the research question and the search terms. The searches were carried out from the inception of the databases to 15 November 2022. Search terms including synonyms and medical subject headings were combined using Boolean operators (OR/AND). Results: Seven studies were included in the review and four themes were identified, including incidence of diabetes; cardiovascular risk factors and other disease risks; indicators of obesity and indicators of environmental sustainability. Two studies examined the association between the PHD and the incidence of type 2 diabetes and found that high adherence to the reference diet (EAT-Lancet reference diet) was correlated with a lower incidence of type 2 diabetes. High adherence to the PHD was also associated with some cardiovascular risk factors and environmental sustainability. Conclusion: This systematic review has shown that high adherence to the PHD is associated with a reduced risk of type 2 diabetes and may be associated with a lower risk of subarachnoid stroke. In addition, an inverse relationship was found between adherence to the PHD and markers of obesity and environmental sustainability. Adherence to the reference diet was also associated with lower values of some markers of cardiovascular risk. More studies are needed to fully examine the relationship between the planetary health diet, type 2 diabetes and its related conditions.

## 1. Introduction

Diabetes prevalence is on the rise globally and about 422 million people are reported to have the condition [1]. The most common form of diabetes is type 2 diabetes and most of those affected by the condition live in low- and middle-income countries, although its impact is felt in countries of all income levels [1]. Diabetes is usually associated with different complications such as kidney dysfunction, cardiovascular disease, retinopathy and stroke, and there is evidence that about 1.5 million deaths per year, worldwide, have been directly associated with diabetes [1,2].

The use of nutritional interventions has been proposed as an effective strategy for reducing the risk of type 2 diabetes and its related complications. Recently, the EAT-Lancet commission, drawing on the principles of planetary health, proposed the reference diet (EAT-Lancet reference diet) or the planetary health diet [3,4]. This is against the backdrop of the role of diet and the effect of current food production systems on environmental sustainability. Therefore, the planetary health diet demonstrates the significant role of diet in linking human health with the need to sustain the environment and the essence of transforming food systems in order to ensure that the United Nations (UN)’s Sustainable Development Goals (SDGs) and the Paris Agreement are achieved [3,4].

### 1.1. Description of the Planetary Health Diet (PHD)

The association between food systems, climate change and human health has been the subject of intense discussion [5]. The publication of the EAT-Lancet commission’s report on ‘Food in the Arthropocene: the EAT-Lancet Commission on healthy diets from sustainable systems’ in 2019 was aimed at providing a global solution for dietary and planetary sustainability [4,6]. The commission recommended a transformation in food systems globally, that would ensure that boundaries and estimates were set with respect to what foods should be eaten by humans and the types of food that should be grown so as to sustain human health and the environment [6].

It has been reported that when this eating pattern is adopted, it will reduce global food waste, improve the efficient use of agricultural resources and promote environmental sustainability [7]. This is particularly important as presently global food systems are a threat to the environment and to human health [8]. Therefore, it is important to move to diets that are sustainable in order to meet the SDGs of the UN [8]. The UN’s SDGs are aimed at ending inequality, climate change, environmental degradation and poverty and ensuring the sustenance of the environment [5,9]. For example, the SDG 12 outlines the need for responsible consumption and production, while Goal 13, which relates to climate action, recommends ‘Acting now to stop global warming’, as the global emissions of carbon dioxide have increased by about 50% since 1990 [9]. The impact of agricultural production on greenhouse gases is significant and it has been estimated that about 25 to 30% of emissions of greenhouse gases globally are contributed to by the agricultural sector [8].

The EAT-Lancet diet is primarily a plant-based diet which has limited amounts of sugar, saturated fat and animal-based products, but is rich in vegetables, whole grains, nuts, fruits and legumes [8]. Furthermore, it has been suggested that the planetary health plate should be made up of fruits and vegetables (50%) and the remaining half of the plate should consist mainly of plant protein sources, whole grains, unsaturated plant oils and limited amounts of animal protein sources [3]. The PHD is similar to the Mediterranean diet which during the 1950s and 1960s was consumed among the people living in the Mediterranean Basin [10]. The similarity is due to the fact that the Mediterranean diet is rich in vegetables, fruits, nuts, legumes, cereals and olive oil, is moderate in the amounts of fish and is low in meat and processed meat [10]. However, there are also differences between the two diets. According to Pérez-Martíneza et al. [11], the animal protein sources including fish and red meat in the Mediterranean diet which generate significant amounts of greenhouse gases will have to be reduced in order to adapt the Mediterranean diet towards the new planetary model. This is particularly important in fish sourced from trawl fishing and recirculation fishing [11].

There is evidence that foods that are plant-based and with limited animal sources can promote health and improve the environment [3]. The principle of the planetary health diet which is applied universally can be adapted and applied locally [3]. Therefore, the planetary health diet should take into consideration the geography, culture and demography of the population and community [3].

The move towards healthy plant-based diets is more sustainable and could help to reduce the impact on the environment and health compared to Western diets which have high amounts of animal products and are often associated with an increased risk of chronic diseases including type 2 diabetes, obesity and cardiovascular disease [8,12,13].

### 1.2. Why It Is Important to Conduct This Review

A healthy diet should promote health, including mental, physical and social wellbeing and meaning more than the absence of disease [3]. However, there is no worldwide agreement in relation to what constitutes a healthy diet and a food production system that is sustainable [3]. While the goals of limiting the burden of non-communicable diseases which have been linked to an unhealthy diet and the drive towards sustainable food systems by the EAT-Lancet report are commendable, the limitations of the approach have been highlighted [14]. According to Verkerk [6], some sections of the EAT-Lancet report are broadly agreed upon, but the author noted that there were areas of weakness, controversy, uncertainty and limitations.

Bäck et al. [15] compared the EAT-Lancet reference diet food group targets with Finnish pre-schoolers’ food consumption. It was concluded that Finnish pre-schoolers should consume more foods that are plant-based such as nuts, vegetables and whole grains in order to meet the EAT-Lancet targets and achieve diets that are more sustainable [15]. In addition, Cacau et al. [16] developed the PHD index (PHDI) based on the proposed EAT-Lancet reference diet (the planetary health diet) and concluded that the PHDI score was related to high dietary quality and reduced carbon footprint overall, after age and sex were adjusted for.

The EAT-Lancet planetary health reference (PHR) diet was compared to the Australian dietary guidelines (ADG) diet, and it was found that while the ADG diet met all of the relevant reference values for the 22 nutrients that were examined, the PHR diet did not meet the requirements for calcium [17]. In addition, the PHR diet and the ADG diet were lower than the average Australian diet with respect to the environmental impact score by 31% and 46%, respectively [17].

Sharma et al. [18] compared patterns of consumption of food in India from seven income groups, sectors and regions with the EAT-Lancet reference diet. The results showed that it was essential to promote efforts aimed at supporting food systems that will ensure that diets which are healthy and sustainable are more acceptable, affordable and accessible [18]. To our knowledge, there has been no systematic review conducted to assess the relationship between the PHD with the risk of type 2 diabetes and its associated complications. The systematic review by Kowalsky et al. [5] was aimed at identifying the co-benefits for the environment and health that can be derived from a sustainable diet.

Research question: what is the association between the planetary health diet and the risk of developing type 2 diabetes and its related complications?

Aim: to examine the association of the planetary health diet with the risk of type 2 diabetes and its related complications.

## 2. Method

The preferred reporting items for systematic reviews and meta-analyses (PRISMA) [19] were used to conduct this systematic review.

Type of intervention: EAT-Lancet diet or planetary health diet

Outcomes of interest:

Primary outcomes:

- Incidence of diabetes

Secondary outcomes:

- Cardiovascular risk factors: total cholesterol, low-density lipoprotein (LDL) cholesterol, high-density lipoprotein (HDL) cholesterol, triglycerides, blood pressure.
- Obesity indicators: body mass index (BMI), waist circumference.
- Indicators of environmental sustainability: greenhouse gas emission, land use (LU).

### 2.1. Search Strategy

Searches were conducted in health sciences research databases through EBSCOHost. The health sciences research databases encompass a range of other databases including Academic Search Premier, MEDLINE, APA PsycInfo, CINAHL Plus with Full Text, Psychology and Behavioral Sciences Collection and APA PsycArticles databases. Searches were also carried out in EMBASE and Google Scholar and the reference lists of articles were searched for the relevant literature.

Searches were carried out using the population, intervention, comparator and outcomes (PICO) framework to define the research question and highlight the search terms (Table 1). The searches were conducted from the inception of the databases to 15 November 2022 and carried out by one researcher (O.O), while a second researcher (O.O.O) repeated the process. Search terms including synonyms and medical subject headings (MeSH) were combined using Boolean operators (OR/AND). Duplicates were removed using EndNote (Analytics, Philadelphia, PA, USA).

### 2.2. Data Collection

#### 2.2.1. Criteria for Inclusion

Prospective cohort studies and cross-sectional studies were included in the review. Studies involving participants without type 2 diabetes, cardiovascular disease or stroke at baseline were also included. In addition, the age of included participants in the studies was 15 years and above.

#### 2.2.2. Criteria for Exclusion

Patients with diabetes, cardiovascular disease or stroke at baseline and children were excluded from the review. Furthermore, feasibility studies and letters or correspondence written to editors were also excluded.

**Figure 1:**
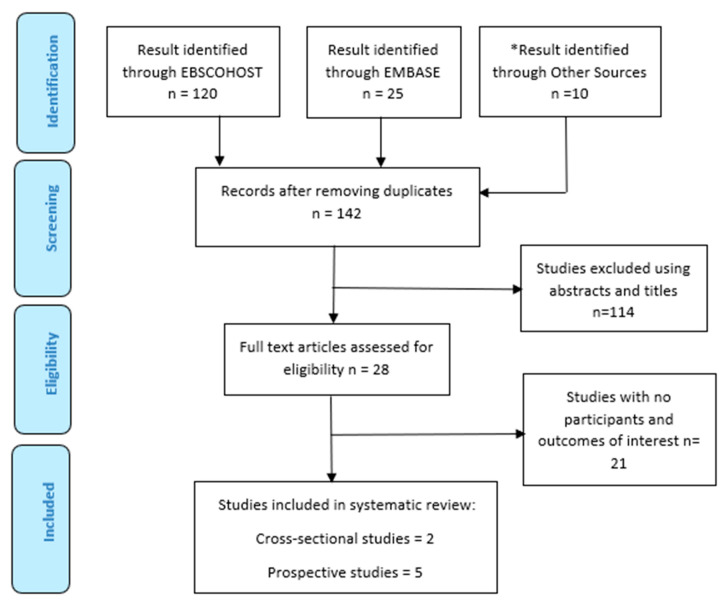
The PRISMA flow chart of included studies. * Other Sources refer to Google Scholar and the reference list of articles.

The PRISMA flow chart (Figure 1) shows the articles included following screening based on the inclusion and exclusion criteria.

### 2.3. Data Extraction and Analysis

The data were extracted from the articles by one researcher (Y.J.) and cross-checked by the other researchers (O.O.; O.O.O.; X.W.). The characteristics and description of the studies included, for example, citation/country of study and year of publication, the aim of the study, the research method, the sample size, the age, the follow-up period and adherence to the planetary health diet/related score, and the results/findings were extracted.

Data on high and low adherence to the PHD in the studies were extracted and discussed in the context of the association between the PHD and the risk of type 2 diabetes and its related complications.

The risk of bias in nonrandomised studies of interventions (ROBINS-I) risk assessment tool [20] was used to assess the quality of the prospective studies, while the Joanna Briggs Institute reviewers’ manual [21] was used for the cross-sectional studies. One researcher (Y.J.) evaluated the quality of the studies and this was cross-checked by X.W. 

The overall risk of bias summary for each included outcome was synthesised by O.O. The process involved evaluating specific risk items/domains within each study and across the studies in relation to the included outcomes of interest. For the ROBINS—I risk assessment tool, the risk items evaluated included bias due to measurement outcomes, bias due to missing data and bias in the selection of participants into the study. Each domain assessed using the ROBIN—1 risk assessment tool had a signalling question, explanation/elaboration and response options [20]. The signalling questions enabled judgements to be made about the risk of bias. The response options were either “Yes”; “Probably yes”; “Probably no”; “No” or “No information”. The options in a green colour represented a potential marker for a low risk of bias, while a response in red represented a potential marker for a risk of bias. The responses to the questions were used to assess the risk of bias for the domain [20]. The judgements in relation to the risk of bias were either “Low risk”, “Moderate risk”, “Serious risk” or “Critical risk” of bias [20].

The Joanna Briggs Institute reviewers’ manual is an appraisal checklist for cross-sectional studies and has eight questions with four response options including ‘Yes’; ‘No’; ‘Unclear’ and ‘Non-applicable’ [21]. The questions include whether the criteria for inclusion in the sample were clearly defined, whether outcomes were measured in a valid and reliable way and whether exposure was measured in a valid and reliable way. Based on the response to each of the questions, an overall appraisal was made as to whether to ‘Include’, ‘Exclude’ or ‘Seek further information’ regarding the study.

The overall risk of bias of outcome was defined as being at ‘high risk of bias’ if one or more of the domains assessed within a study had a high risk of bias or if some of the information across the studies at a high risk of bias may have affected the interpretation of the findings.

## 3. Results

Seven studies were included in the review [22,23,24,25,26,27,28]. Two studies were conducted in Brazil [23,24], while one study each was carried out in France [22], Denmark [25], Mexico [26], Germany [27] and the UK [28] (Table 2).

### 3.1. Assessment of Quality of Studies

**Figure 2:**
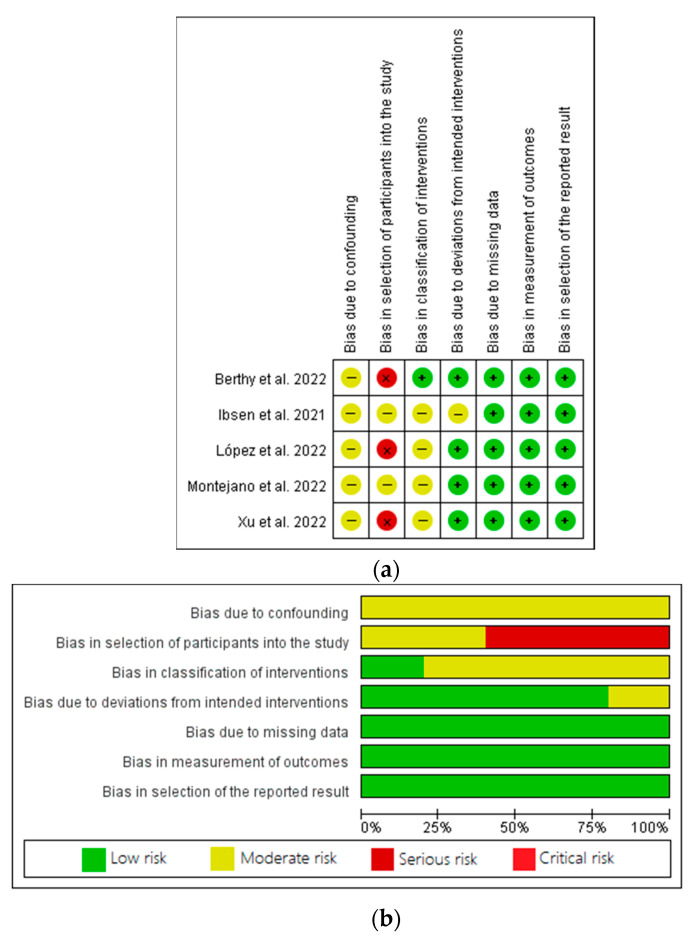
(**a**,**b**): Quality assessment of prospective cohort studies [22,25,26,27,28], according to the Cochrane collaboration standard scheme for bias and ROBINS-I tool.

The five prospective cohort studies were evaluated using the risk of bias in the nonrandomised studies of interventions (ROBINS-I) risk assessment tool (Figure 2a,b). The domains assessed were confounding factors, selection of participants, classification of interventions, deviations from intended interventions, missing data, measurement of outcomes and the selection of reported results.

Three studies were classified as having a ‘serious’ risk of bias in the selection of participants, while the other two studies were classified as having a ‘moderate’ risk of bias (Figure 2a,b). In the domain due to confounding factors, all of the studies had a moderate risk of bias, while with respect to the classification of interventions, four studies had a moderate risk of bias and one study had a low risk of bias. In the domain due to deviations from intended interventions, one study had a moderate risk of bias, and the others had a low risk of bias. With respect to the three remaining domains, all studies had a low risk of bias.

**Figure 3:**
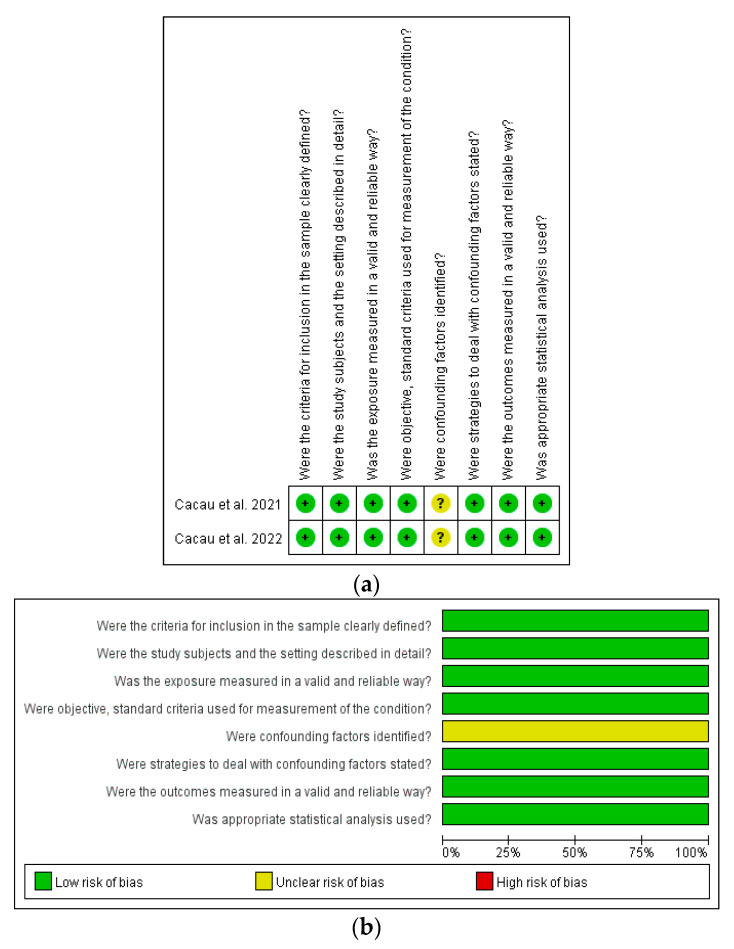
(**a**,**b**): Quality assessment of cross-sectional studies [23,24], according to the Joanna Briggs Institute reviewers’ manual.

The two cross-sectional studies were assessed using the Joanna Briggs Institute reviewers’ manual (Figure 3a,b). The two studies were rated as having ‘low risk’ in seven of the eight items on the checklist. The checklist items rated as ‘unclear risk’ were mostly related to ‘whether confounders were identified’.

### 3.2. Summary Assessments of the Risk of Bias for Included Outcomes

Based on the criteria for defining the high risk of bias for included outcomes, the overall risk of bias with respect to the incidence of diabetes was high across the studies [26,28], and this may weaken the confidence in the findings. In relation to cardiovascular risk factors, other disease risks and indicators of obesity, while the overall risk of bias was high in some studies [22,26,28], it was low across the studies. This would suggest that although there may be a bit of doubt about the results, the bias is unlikely to significantly change the findings. The overall risk of bias for environmental sustainability was low within a study and across the studies. Therefore, the bias is not likely to significantly change the results.

### 3.3. The Systematic Review Identified Four Distinct Themes

#### 3.3.1. Incidence of Diabetes

Two studies [26,28] examined the association between the planetary health diet and the incidence of type 2 diabetes. Lopez et al. [26] found that higher adherence to the EAT-Lancet reference diet score was correlated with a lower incidence of type 2 diabetes. Participants who adhered to recommendations relating to legumes, fish and red meat were associated with a lower incidence of type 2 diabetes compared to those who did not adhere to the recommendations. On the other hand, meeting the added sugars and dairy recommendations was associated with higher type 2 diabetes incidence compared to not meeting the recommendations [26].

In a separate study, Xu et al. [28] noted that a one-point increase in the EAT-Lancet diet pattern score reported by participants was associated with a decrease of 6% in type 2 diabetes risk. Therefore, it was concluded that significant adherence to the EAT-Lancet diet pattern will no doubt contribute to lowering the risk of type 2 diabetes [28].

#### 3.3.2. Cardiovascular Risk and Other Disease Risks

Berthy et al. [22] did not find any significant relationship between cardiovascular disease risk and the EAT-Lancet diet. It was also reported that among low drinkers of alcohol, there was a significant association between the EAT-Lancet diet and cardiovascular disease. 

According to Cacao et al. [24], the participants who adhered more to the EAT-Lancet diet (planetary health diet index, fifth quintile) were found to have lower values for low-density lipoprotein cholesterol (LDL-c), diastolic blood pressure, systolic blood pressure, total cholesterol and non-HDL cholesterol. However, there was no association between the EAT-Lancet diet and triglycerides, high-density lipoprotein cholesterol (HDL-c) and homeostatic model assessment for insulin resistance (HOMA-IR) [26].

Ibsen et al. [25] found that those participants who in midlife adhered to the EAT-Lancet diet were associated with a reduced risk of subarachnoid stroke, and the alternate healthy eating index was associated with a reduced risk of total stroke, including intracerebral haemorrhage and ischemic stroke.

The results of adherence to the dietary index score which was developed from the EAT-Lancet reference diet showed that the highest tertile of the dietary index score was inversely associated with the intake of protein, added sugars and cholesterol, but positively associated with fibre intake [27]. Montejano et al. [27] did not find any associations between the dietary index score and cardiometabolic risk markers.

#### 3.3.3. Indicators of Obesity

It has been reported that there is an indirect association between the EAT-Lancet diet pattern score and the risk of developing type 2 diabetes [28]. In particular, Xu et al. [28] found that 44% of the association of the EAT-Lancet diet pattern score and type 2 diabetes was due to body mass index, while 40% of the association was as a result of waist circumference. Berthy et al. [22] also found that the association between the EAT-Lancet diet and cardiovascular disease was mostly mediated by body mass index.

There was an inverse relationship between participants that adhered to the PHD and indicators of obesity [23]. For example, those with high adherence to the PHD had lower body mass index and waist circumference and were 24% less likely to be overweight or obese [23]. These individuals were also 14% and 27% less likely to have increased waist circumference or significantly increased waist circumference, respectively, compared with those who had lower adherence [23].

There was evidence of inverse associations between the dietary index score and anthropometric markers, including body mass index during young adulthood [27].

#### 3.3.4. Indicators of Environmental Sustainability

The dietary index score was found to be inversely associated with greenhouse gas emissions and land use [27]. For example, Tertile 1 was 6.48 compared to Tertile 3, which was 5.85 kg of carbon dioxide equivalents/2500 kcal (*p* < 0.001) [27]. For land use, Tertile 1 was 8.24 compared to Tertile 3, which was 7.16 m^2^ × y/2500 kcal (*p* < 0.001) [27]. 

## 4. Discussion

This systematic review has shown that high adherence to the EAT-Lancet reference diet or the PHD was associated with a reduced risk of type 2 diabetes and may be associated with a lower risk of subarachnoid stroke. Evidence of these could be found in some of the prospective studies [25,26,27]. 

With respect to cardiovascular risk, while two prospective studies, Berthy et al. [22] and Montejano et al. [27], did not find any significant association between the PHD and markers of cardiovascular risk, the cross-sectional study by Cacao et al. [24] found that adherence to the reference diet was associated with lower values for low-density lipoprotein cholesterol, diastolic blood pressure, total cholesterol, systolic blood pressure and high-density lipoprotein cholesterol. The differences in the findings between the prospective and cross-sectional studies in relation to cardiovascular risk factors could be due to the fact that these are different levels of evidence.

Cacau et al. [23] also found an inverse relationship between the PHD and markers of obesity, while the prospective study by Montejano et al. [27] showed an inverse relationship between the PHD and environmental sustainability. 

The findings of this review with respect to the association between the risk of diabetes, body mass index and some cardiometabolic markers appear to confirm the results of a previous study conducted by Knuppel et al. [29]. Knuppel et al. [29] found that high adherence to the EAT-Lancet diet was related to the lowering of the risk of diabetes by 59%, a reduction of about 1.4 kg/m^2^ in body mass index in sub-samples, while there were significant reductions in non-HDL cholesterol and systolic blood pressure compared with low adherence. 

It would appear that the lowering of the risk of type 2 diabetes and some of the markers of cardiovascular risk by the PHD may be through different mechanisms, including the direct effect of the food components on glycaemic control and the indirect effects through the mediation of body mass index and waist circumference. For example, Xu et al. [28] noted that the PHD contributes to the prevention of type 2 diabetes even without influencing changes in BMI or waist circumference. In addition to the individual effect of each food component, the combined effects of the different food groups in the PHD bring improved benefits compared with that of each isolated nutrition [28]. However, mediation analysis carried out by Xu et al. [28] found that the risk of type 2 diabetes based on adherence to the PHD was mediated significantly by body mass index and waist circumference. 

According to Hemler and Hu [12], healthy plant-based diets are more sustainable and have also been associated with lowering the risk of chronic diseases, including type 2 diabetes, obesity and cardiovascular disease, compared with Western diets which are high in animal products. The PHD provides a dietary outline that has a global outlook, encompassing eight food groups based on global foods [15]. The food groups include vegetables, whole grains, fruits, dairy foods, starchy vegetables/tubers, protein sources (including meat and alternatives), added sugars and added fats [6,15]. 

The PHD proposes average daily intakes for adults from the eight food groups, including having limited intakes of starchy vegetables, zero to minimal amounts of meat, very limited intakes of saturated fats, allowance for added sugars, limiting the intake of palm oil to 6.8 g/day or 2.4% of one’s daily energy and having more energy allowance for sugar than for meat [6]. In addition, the PHD proposes the replacement of animal fats with plant oils and whole grains, contributing 32% of one’s daily energy, while fruits and vegetables should contribute 8% of one’s daily energy [6]. The inclusion of a high percentage of whole grains in the PHD would suggest the presence of high fibre in the diet, as the use of whole grain foods is a practical way of increasing fibre intake [30]. This is particularly important as the mechanisms through which a healthy plant-based diet could lower the risk of type 2 diabetes include being rich in dietary fibre, unsaturated fatty acids, antioxidants and micronutrients, and low in saturated fat [31]. 

The use of diets that are high in fibre and are vegetable-based over a long period may provide effects that are beneficial through enhancing the composition of gut/faecal microbiota and may improve glycaemia, inflammation and dyslipidaemia [32]. Furthermore, there is evidence to confirm the beneficial effects of non-starch polysaccharides (NSPs) in relation to colonic function, including stool weight/mass and transit time, and the lowering effect of soluble fibre with respect to LDL cholesterol and total cholesterol [33].

Soluble fibres have been reported to delay the emptying of the stomach and slow down the entry of glucose into the blood stream, and therefore reduce a postprandial rise in blood glucose [34,35]. In addition, an alteration in the production of glucagon-like peptide-1 (GLP-1) that is produced in the gut and is involved in glucose metabolism may be influenced by soluble fibres [34].

Energy and blood glucose levels as well as the effectiveness of pharmacological interventions have been reported to be influenced by the gut microbiome [36,37]. In particular, the fermentation of most soluble fibres by microbes in the gut leads to short-chain fatty acid (SCFA) production that may influence the levels of serum glucose and insulin [37,38]. SCFAs can regulate lipid and glucose metabolism by activating SCFA receptors in the liver and adipose tissue [2,39,40]. Acetate can directly and indirectly regulate one’s appetite and promote GLP-1 and peptide YY production, which are hormones that are produced from the L-cells of the intestine and which can suppress one’s appetite [35,41].

Stubbendorff et al. [8] found that those who adhered highly to the PHD had a total energy intake that was lower compared with those with low adherence. There is evidence that obesity and being overweight are risk factors in the development of type 2 diabetes; therefore, weight loss can reverse the pathophysiology of the disease and improve glycaemic control [42]. Findings from the Diabetes Remission Clinical Trial demonstrated that reductions in the weight of individuals with type 2 diabetes who were overweight and obese led to remission [42].

It would appear that there is a direct link between obesity-related insulin resistance and the adipose tissue expression of tumour necrosis factor (TNF-α) [43]. This is because the levels of TNF-α messenger ribonucleic acid (mRNA) and the production of TNF-α in human skeletal muscle are raised in patients with type 2 diabetes or insulin resistance [43].

Raised levels of free fatty acids (FFAs) have been associated with peripheral and hepatic insulin resistance [44]. Elevated levels of FFA and intracellular lipid may impair insulin signalling, which could affect the translocation of GLUT-4 from the cell cytoplasm to the cell membrane, causing a reduction in glucose transportation [44]. This can also influence glycogen synthesis, leading to a loss of glucose homeostasis [2]. In particular, the suppression of the transportation of glucose from the extracellular compartment to the intracellular compartment due to insulin resistance leads to a reduction in the synthesis of muscle glycogen and glycolysis, causing hyperglycaemia [44].

## 5. Limitations

There were seven studies included in the systematic review and there were limited studies that addressed the association between the PHD and the chronic conditions of interest. The high risk of bias in some of the studies [22,26,28] may weaken the confidence in the findings in relation to the incidence of diabetes, while in other outcomes, such as cardiovascular risk factors and indicators of obesity, although there may be a bit of doubt about the results, the bias is unlikely to significantly change the findings. Therefore, although the current review provides a basis for evaluating the association of the PHD with type 2 diabetes and its related complications, the review should be seen as exploratory and more studies are needed to fully assess the relationship been the PHD, type 2 diabetes and its related conditions. 

## 6. Conclusions

This systematic review has revealed that high adherence to the planetary health diet was associated with a reduced risk of type 2 diabetes and may be associated with a lower risk of subarachnoid stroke. Furthermore, the review found an inverse relationship between adherence to the planetary health diet and markers of obesity and environmental sustainability. Adherence to the reference diet was associated with lower values of some markers of cardiovascular risk. More studies are required to fully assess the relationship been the planetary health diet, type 2 diabetes and its related conditions.

## Figures and Tables

**Table 1:** Search terms using the PICO framework.

Patient/Population	Intervention	Outcome (Primary)	Combining Search Terms
Patients with diabetes and its associated complications	EAT-Lancet diet OR Planetary health diet		
Diabetes mellitus, type 2 OR Diabetes complications OR Patients with diabetes OR Diabetes mellitus OR Type 2 diabetes OR Diabetes OR Stroke OR Cardiovascular disease OR Kidney dysfunction	EAT-Lancet diet OR Planetary health diet OR Evolution planetary OR EAT-Lancet diet planetary OR Sustainable diet		Column 1 AND Column 2

**Table 2:** The description and characteristics of the included studies.

Author/Country of Study	Aims/Objectives	Study Type	Sample Size	Age at Baseline, Y	Number of Females, n (%)	Follow-Up Time, y	Exposure	Main Findings
Berthy et al. [22], France	To investigate the relationship between adherence to the EAT-Lancet diet and major chronic disease risks.	Prospective cohort study.	n = 62,382	51.0 ± 10.2	47,286 (75.8%)	8.1	The ELD-I based on 24 h dietary records.	• The association between a higher adherence to the EAT-Lancet diet and cancer and CVD risks combined (*p* = 0.19) and separately was not significant (*p* = 0.18, *p* = 0.60). • Among low consumers of alcohol (<20 g/d), high ELD-I was negatively associated with cancer and CVD risks combined (HR_Q5vs_._Q1_: 0.86; 95% CI: 0.73, 1.02; *p* = 0.02). • Among females, higher adherence to the EAT-Lancet diet was associated with a lower risk of cancer (HR_Q5vs_._Q1_: 0.89; 95% CI: 0.75, 1.05; *p* = 0.03), but was largely attenuated by BMI.
Cacau et al. [23], Brazil	To examine adherence to the PHDI and obesity outcomes (i.e., BMI, WC) using baseline data in the ELSA-Brasil.	Cross-sectional analysis of multi-centre and ongoing cohort study.	n = 14,515	35–74	7923 (54.58%)	Not Applicable	The PHDI.	• There was an inverse relationship between obesity indicators and adherence to the PHDI. • High adherence to the PHDI was found in individuals with lower BMI values. • Participants with the higher PHDI scores were less likely to be overweight (*p* < 0.001) and obese (*p* < 0.001) and less likely to have abdominal obesity (*p* < 0.001) than those with lower adherence.
Cacau et al. [24], Brazil	To assess the relationship between adherence to the EAT-Lancet diet with cardiometabolic risk profile.	Cross-sectional baseline data from the Brazilian longitudinal cohort study.	n = 14,155	35–74 adults (34–59 years): 11,216 (79.24%)	7746 (54.72%)	Not Applicable	The PHDI was adapted from recommendations of the reference diet proposed by the EAT-Lancet Commission.	• Lower values of blood pressure, total cholesterol, LDL-c and non-HDL-c were significantly associated with higher adherence to the EAT-Lancet diet. • Better cardiovascular health was also associated with higher adherence to the EAT-Lancet diet.
Ibsen et al. [25], Denmark	To examine the relationship between adherence to the EAT-Lancet diet and risk of stroke and subtypes of stroke in a Danish population. For comparison, the AHEI was also investigated.	Prospective cohort study.	n = 55,016	56.0 (51.0, 63.0)	28,808 (52.36%)	15	The EAT-Lancet diet.	• Lower risk of stroke was associated with adherence to the EAT-Lancet diet. However, this was not significant statistically (highest versus lowest adherence: HR: 0.91; 95% CI: 0.76–1.09). • For stroke subtypes, a lower risk of subarachnoid haemorrhage was associated with adherence to the EAT-Lancet diet (HR: 0.30; 95% CI: 0.12–0.73). • Adherence to the AHEI was related to a lower risk of total stroke, mainly ischemic stroke and intracerebral haemorrhage.
López et al. [26], Mexico	To explore the relationship between the EAT-HRD and the incidence of T2D.	Prospective cohort study.	n = 74,671	42.1 ± 7.1	74,671 (100%)	2.2 (inter-quartile range: 1.8, 4.3)	The EAT-HRD score.	• Lower incidence of T2D was associated with a higher adherence to the EAT-HRD score (HR: 0.90; 95% CI: 0.75, 1.10). • Adhering to the red meat (HR: 0.79; 95% CI: 0.63, 0.99), legumes (HR: 0.92; 95% CI: 0.84, 0.99) and fish (HR: 0.92; 95% CI: 0.85, 1.00) recommendations was associated with lower T2D incidence compared to those who did not meet the EAT-HRD recommendations. • An increased incidence of T2D was associated with meeting the EAT-HRD recommendations for dairy (HR: 1.12; 95% CI: 1.04, 1.21) and added sugars (HR: 1.11; 95% CI: 1.02, 1.21).
Montejano et al. [27], Germany	To study the association between the DI score (reflects adherence to the EAT-Lancet reference diet) and anthropometric parameters and cardiometabolic biomarkers.	Prospective cohort study.	n = 298	16.7	143 (47.99%)	3.01 (range: 1.58–32.2)	The DI score based on 3 d weighted dietary records to measure adherence to the EAT-Lancet.	• There was an inverse relationship between the DI score during adolescence and BW (*p* = 0.009) and BMI (*p* = 0.015). • No associations between the DI score and cardiometabolic risk markers (FPG, total cholesterol, LDL-c, HDL-c, TGs, SBP and DBP) were found (all *p* ≥ 0.05).
Xu et al. [28], UK	To examine the relations between the risk of T2D and EAT-LDP.	Prospective cohort study.	n = 59,849	55.9 ± 8.1	34,512 (57.67%)	10	The EAT-LDP score.	• A lower risk of T2D was contributed to by higher adherence to EAT-LDP. • Compared with the group with the lowest EAT-LDP score (1st tertile), the risk of T2D decreased in the highest group (HR: 0.81; 95% CI: 0.72–0.90). • A 6% decrease in the risk of T2D was associated with participants who reported a one-point increase in the diet score (HR: 0.94; 95% CI: 0.91–0.97).

Abbreviations: AHEI: alternate healthy eating index; BMI: body mass index; BW: body weight; CKD: chronic kidney disease; DI: dietary index; DBP: diastolic blood pressure; EAT-LDP: EAT-Lancet diet pattern; ELD-I: EAT-Lancet diet index; EAT-HRD: EAT-Lancet healthy reference diet; ELSA-Brasil: Brazilian longitudinal study of adult health; FPG: fasting plasma glucose; HDL-c: HDL cholesterol; HR: hazard ratio; LDL-c: LDL cholesterol; PHDI: planetary health diet index; SBP: systolic blood pressure; T2D: type 2 diabetes; TGs: triglycerides; WC: waist circumference.

## Data Availability

The review was based on secondary data analysis of publicly available data.
